# Mitochondrial genome of *Babesia orientalis*, apicomplexan parasite of water buffalo (*Bubalus babalis*, Linnaeus, 1758) endemic in China

**DOI:** 10.1186/1756-3305-7-82

**Published:** 2014-02-28

**Authors:** Lan He, Ying Zhang, Qing-Li Zhang, Wen-Jie Zhang, Hui-Hui Feng, Muhammad Kasib Khan, Min Hu, Yan-Qin Zhou, Jun-Long Zhao

**Affiliations:** 1 State Key Laboratory of Agricultural Microbiology, College of Veterinary Medicine, Huazhong Agricultural University, Wuhan, Hubei, 430070, China; 2 Key Laboratory of Animal Epidemical Disease and Infectious Zoonoses, Ministry of Agriculture, Huazhong Agricultural University, Wuhan, Hubei, 430070, China

**Keywords:** *Babesia orientalis*, Mitochondrion, Mitochondrial genome, Phylogeny

## Abstract

**Background:**

Apicomplexan parasites of the genus *Babesia*, *Theileria* and *Plasmodium* are very closely related organisms. Interestingly, their mitochondrial (mt) genomes are highly divergent. Among *Babesia*, *Babesia orientalis* is a new species recently identified and specifically epidemic to the southern part of China, causing severe disease to water buffalo. However, no information on the mt genome of *B. orientalis* was available.

**Methods:**

Four pairs of primers were designed based on the full genome sequence of *B. orientalis* (unpublished data) and by aligning reported mt genomes of *B. bovis*, *B. bigemina*, and *T. parva*. The entire mt genome was amplified by four sets of PCR. The obtained mt genome was annotated by aligning with published apicomplexan mt genomes and Artemis software v11. Phylogenetic analysis was performed by using *cox1* and *cob* amino acid sequences.

**Results:**

The complete mt genome of *B. orientalis* (Wuhan strain) was sequenced and characterized. The entire mt genome is 5996 bp in length with a linear form, containing three protein-coding genes including cytochrome *c* oxidase I (*cox1*), cytochrome b (*cob*) and cytochrome *c* oxidase III (*cox3*) and six rRNA large subunit gene fragments. The gene arrangement in *B. orientalis* mt genome is similar to those of *B. bovis*, *B. gibsoni* and *Theileria parva*, but different from those of *T. orientalis*, *T. equi* and *Plasmodium falciparum*. Comparative analysis indicated that *cox1* and *cob* genes were more conserved than *cox3*. Phylogenetic analysis based on amino acid sequences of *cox1*, *cob* and *cox1* + *cob*, respectively, revealed that *B. orientalis* fell into *Babesia* clade with the closest relationship to *B. bovis*.

**Conclusions:**

The availability of the entire mt genome sequences of *B. orientalis* provides valuable information for future phylogenetic, population genetics and molecular epidemiological studies of apicomplexan parasites.

## Background

Mitochondria are essential organelles within cells and are responsible for energy transduction, metabolism, cell growth and survival [1-3]. Inside mitochondria, there is a genome called mitochondrial (mt) genome. Mt genomes are present in almost all eukaryotic cells and have remarkable variations in size, structure, and organization [4-6]. The largest mt genome has been found in muskmelons with an estimated size of 2400 kb [7-9]. The smallest mt genome of only 6 kb in length has been reported in an apicomplexan parasite (*Plasmodium*) [10,11].

The structure of mt genome contains two major types, the linear form and the circular form. The circular forms are usually present in animal mt genomes with the size ranging from 15 kb to 20 kb, containing 12-13 protein-coding genes, 22 transfer RNA (tRNA) genes and two ribosomal RNA (rRNA) genes, and gene arrangements in the genomes are extremely stable [12]. The linear forms have been documented in many apicomplexan parasites, including *Plasmodium*, *Babesia, Theileria* and *Eimeria*[5,10,11,13]. Compared with animal mt genomes, the mt genomes of apicomplexan parasites encode only three protein-coding genes (cytochrome c oxidase subunits I [*cox1*] and III [*cox3*], and cytochrome b [*cob*]) and six fragments of large subunit rRNA genes [14]. The gene arrangements are also different among animal mt genomes and apicomplexan parasites mt genome.

Meanwhile, in apicomplexan parasites, mitochondrial protein-coding genes have been extensively used as genetic markers for phylogenetic analysis at different taxonomic levels, serving as an ideal model for gene rearrangement, and evolutionary studies [13,15]. Most of the phylogenies of apicomplexan parasites were constructed using *cox1* or *cob* alone, however, in some cases, a combination of *cox1* and *cob* was employed to evaluate the phylogenetic relationships [13,16]. In addition, mt genome sequences are also very valuable for population genetic studies [17-19] as reported in *Plasmodium vivax, Plasmodium knowlesi*, *Trypanosoma cruzi*[20-22].

*B. orientalis* is a tick-borne, intra-erythrocytic protozoan parasite causing buffalo babesiosis characterized by fever, anemia, icterus, haemoglobinuria and high mortality [23,24]. This species was first reported in 1987 and then identified as a new species named *Babesia orientalis* in 1997 [25]. The new species was discovered initially based on the differences in morphology, transmission, pathogenicity and endemic areas, compared to *Babesia bigemina* and *Babesia bovis*[25], and later confirmed by the phylogenetic analysis based on 18S rRNA and heat shock protein 70 (HSP70) genes [26,27]. The disease caused by *B. orientalis* is one of the most important parasitic diseases of buffalo in central and south China, resulting in enormous economic losses [27,28]. In spite of its importance, very limited information was available about this parasite, especially at the molecular level, including mt genome sequences and structures.

In the present study, *B. orientalis* (Wuhan strain) mt genome was determined and annotated. The structure was characterized and compared with those of related species. In addition, the evolution of structural divergence in the apicomplexan mt genomes was discussed.

## Methods

### Parasite cultivation

*Babesia orientalis* (Wuhan strain) was cultivated according to the protocol of He et al. [29]. In brief, two, 1-year-old water buffalo, free of *B. orientalis* infection as confirmed by microscopy and real-time PCR [29], were splenectomized 14 days prior to *B. orientalis* infection. Each buffalo was subcutaneously injected with 4 ml of *B. orientalis*-infected blood (Wuhan strain, percentage parasitized erythrocytes, PPE 1%). Blood samples were collected everyday to monitor the parasitemia until PPE reached 3%.

All the experimental animals were housed, fed and given clean drinking water in accordance with the stipulated rules for the regulation of the administration of affairs concerning experimental animals of P.R. China.

### *B. orientalis* mitochondria DNA sequencing

**Figure 1:**
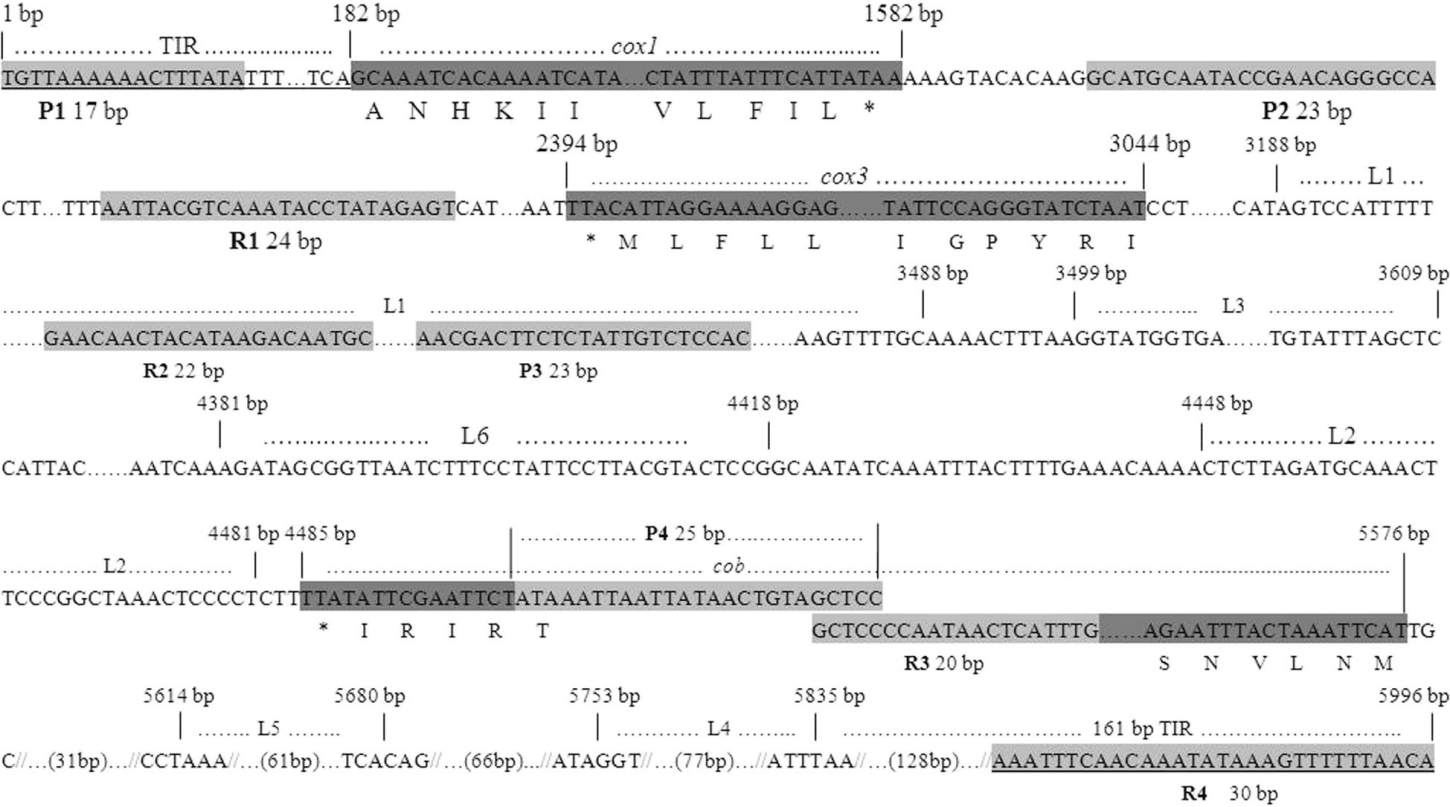
**Schematic representation of the mt genome of *B. orientalis*. **Symbol … and ()in the sequence indicates nucleotide omitted. Primer sequences of P1, P2, P3, P4, R1, R2, R3 and R4 are shown in light grey. Three protein-coding genes *cox*1, *cox*3 and *cob* are shown in dark grey. The first and last six codons of each protein-coding gene are shown below the sequence. Six LSU rRNA genes from L1–L6 are showed. Terminal inverted repeats (TIR) are represented by underlining. The symbolㄧabove the genome sequence indicates the beginning and the end of the gene fragment. The nucleotide position of each gene fragment is shown above the sequences.

The blood from experimentally infected buffalo was collected in EDTA. Parasite genomic DNA was extracted from *B. orientalis*-infected blood using QIAamp DNA Blood Mini Kit (Qiagen, Hilden, Germany) according to the manufacturer’s instructions. The terminal inverted repeat (TIR) regions in the beginning and end of *B. orientalis* mt genome, 1-181 bp and 5835-5996 bp, were obtained by using *B. bovis* mt genome sequence to blast the full genome sequence of *B. orientalis* (unpublished data). Four pairs of primers were designed based on these two mtDNA sequences (1-181 bp and 5835-5996 bp) of *B. orientalis* and by aligning reported mt genomes of *B. bovis* (EU075182 and AB499088), *B. bigemina* (AB499085), and *T. parva* (Z23263 and AB499089) (Table 1 and Figure 1). The entire mt genome was amplified by P1 and R4. In order to confirm the mt genome, four sets of PCR were processed using primers P1 and R1, P2 and R2, P3 and R3, P4 and R4, respectively (Figure 1). Amplified products were purified and then ligated into the pMD19-T vector (TaKaRa Biotechnology), and the recombinant clones were sequenced using the ABI PRISM 377 DNA sequencer by following the manufacturer’s instructions. The vector primers M13 (-47) and M13 (-48), as well as PCR primers, were used for the sequencing of mt genome.

**Table 1:** **Primers used for cloning of *Babesiaorientalis *** **mt genome sequence**

**Primers**	**Sequences**	**Position**
P1	5′-TGTTAAAAAACTTTATA-3′	1-17
R1	5′-ACTCTATAGGTATTTGACGTAATT-3′	1749-1726
P2	5′-GCATGCAATACCGAACAGGGCCA-3′	1592-1617
R2	5′-GCATTGTCTTATGTAGTTGTTC-3′	3246-3225
P3	5′-AACGACTTCTCTATTGTCTCCAC-3′	3368-3390
R3	5′-CAAATGAGTTATTGGGGAGC-3′	5198-5179
P4	5′-ATAAATTAATTATAACTGTAGCTCC-3′	5159-5183
R4	5′-TGTTAAAAAACTTTATATTTGTTGAAATTT-3′	5967-5996

### Gene annotation and sequence analysis

Nucleotide sequences of *B. orientalis* mt genome (GenBank accession no. KF218819) were aligned with published mt genome sequences of *Plasmodium falciparum* (M76611), *B. bovis* (AB499088), *T. parva* (AB499089), and *T. annulata* (NT167255) by MAFFT (version 7) [30,31] with manual corrections. Protein-coding genes were predicted by comparing the previously annotated sequences from these four related species. To identify putative rRNA genes, mt DNA sequences or annotated rRNA gene fragments from the four related species were used as queries by pair-wise comparison (Blastn) in NCBI. The entire *B. orientalis* mt genome was subjected to tRNAscan SE 1.21 (http://lowelab.ucsc.edu/tRNAscan-SE/) using Mito/Chloroplast model option and Nematode Mito model for analyzing the existence of tRNA gene. Results from both models were compared, final annotation was determined according to *B. bovis* mt genome annotation. *B. orientalis* mt genome was also annotated using appropriate mitochondrial codes in Artemis software v11 [32,33].

Nucleotide sequence identities were calculated by pairwise comparison between 13 apicomplexan parasites including *B. orientalis*, another six *Babesia* species, five *Theileria* species and *P. falciparum* for each of the three protein-encoding genes. The data sets of *cox1* (472 aa), *cox3* (229 aa) or *cob* (358 aa) were aligned using MAFFT (version 7) employing the FFT-NS-i algorithm [31,34]. The alignment was manually edited using BioEdit 7.1.11 [35]. The nucleotide identities were determined through BioEdit 7.1.11.

### Phylogenetic analysis

The concatenated amino acid sequences of *cox1*, *cox3* and *cob* from 15 apicomplexan parasites (Table 2) were used for similarity analysis. For phylogenetic analysis, *cox3* was not included, because *cox3* has been presented in the nuclear genome rather than in mt DNA in some species, such as *T. thermophila*. A free-living ciliate, *Tetrahymena thermophila*[36], was used as the outgroup. The best-fit model of *cox1*, *cob*, and *cox1*-*cob* combined nucleotide substitution was determined by JmodelTest 0.1.1 [37] selected by AIC calculations. A general time reversible model with a proportion of invariable sites and a gamma-shaped distribution of rates across sites (TIM1 + I + G) substitution mode was used in PAUP* v4b10 [38] to explore neighbour-joining, parsimony and maximum likelihood methods. MrBayes v3.1.2 [39,40] was used to explore Bayesian phylogeny. Consensus trees were edited in MEGA v4.0.2 [41].

**Table 2:** Mitochondrial genome sequences of apicomplexa parasites used in the present study

**Family**	**Species**	**Size of mt DNA (bp)**	**GenBank accession no.**
Babesiidae	*B. orientalis*	5996	KF218819
	*B. bovis*	6005	EU075182
	*B. bovis*	5970	AB499088
	*B. bigemina*	5924	AB499085
	*B. caballi*	5847	AB499086
	*B. gibsoni*	5865	AB499087
	*B. rodhaini*	6929	AB624357
Theileriidae	*T. parva*	5895	Z23263
	*T. parve*	5924	AB499089
	*T. equi*	8246	AB499091
	*T. orientalis*	5957	AB499090
	*T. annulata*	5905	NT167255
Plasmodiidae	*P. falciparum*	5967	M76611

All water buffalo studies were carried out in compliance with the regulations (No. 5 proclaim of the Standing Committee of Hubei People's Congress) approved by the Standing Committee of Hubei People's Congress, P. R. China. The animal protocols were approved by Laboratory Animals Research Centre of Hubei province and the ethics committee of Huazhong Agricultural University (Permit number: 4200696657).

## Results and discussion

### Characterization of *B. orientalis* mt genome

The full length mt genome of *B. orientalis* was amplified by P1 and R4, and a 5996 bp fragment was obtained. In order to confirm the mt genome sequence, four overlapping fragments were amplified by using primer sets P1-R1, P2-R2, P3-R3 and P4-R4 with expected sizes of 1749 bp, 1655 bp, 1831 bp and 838 bp, respectively. These four overlapping fragments covered the entire genome of *B. orientalis* (Figure 1). Each of the PCR products were then cloned into pMD19-T vector, and sequenced. A 5996 bp mt genome sequence of *B. orientalis* (Wuhan strain) was obtained by assembling all the sequenced fragments.

**Figure 2:**
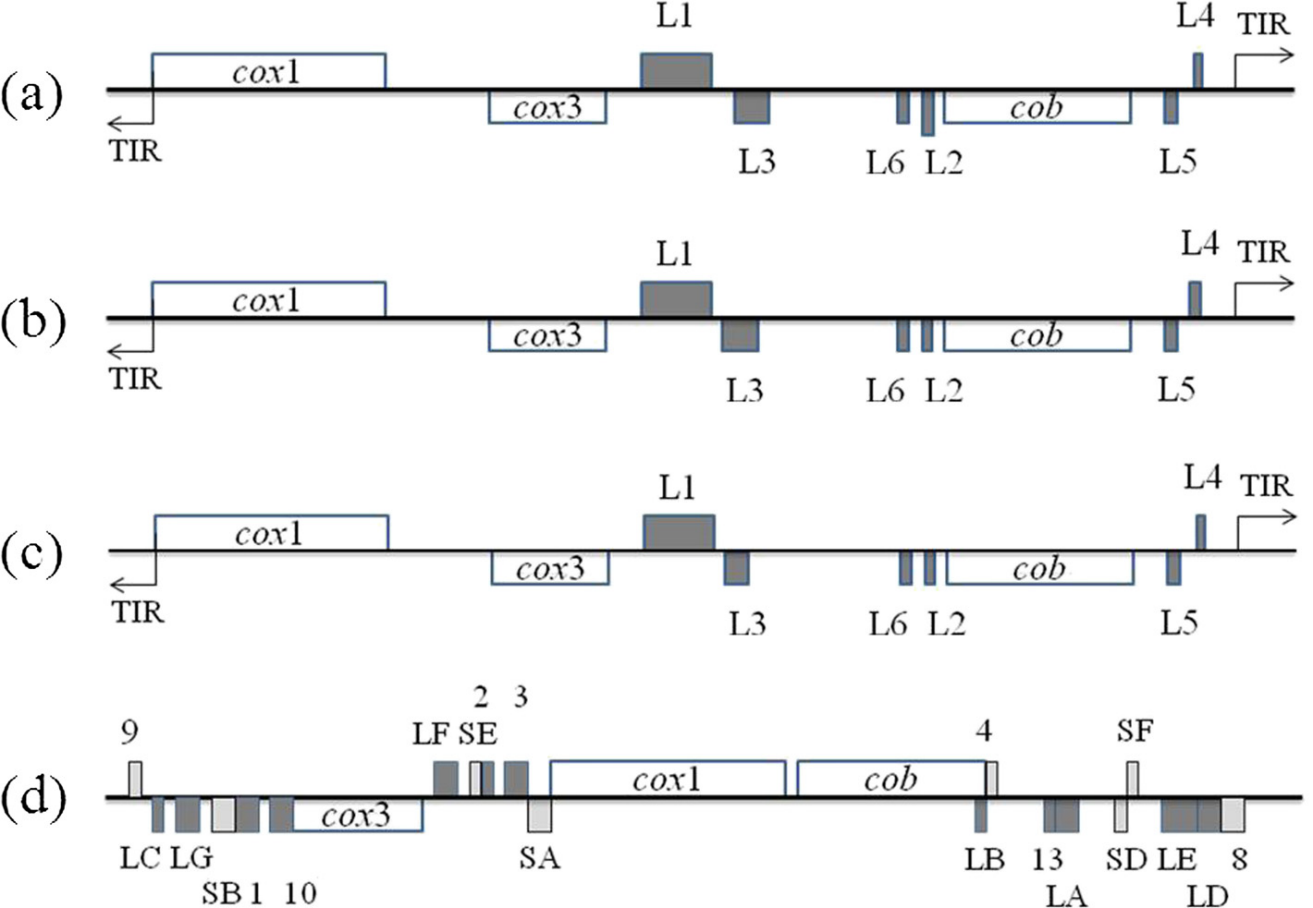
**Schematic structure of mitochondrial genomes of *Babesia orientalis *(KF218819) (a) and three apicomplexan parasites including (b) *B. bovis *(AB499088), (c) *Theileriaparva *(Z23263) and (d) *Plasmodium falciparum *(M76611).** Gene names above the bold line are transcribed from left to right, and those below are transcribed from right to left. White boxes indicate protein-coding genes, *cox*1 (cytochrome *c* oxidase I), *cox*3 (cytochrome *c* oxidase III), and *cob* (cytochrome *b*). Fragments of LSU (L1 – L6, LA – LG, 1, 2, 3, 10 and 13) and SSU (SA, SB, SD – SF, 4, 8 and 9) rRNA genes are shown by dark and light gray boxes. TIR represents terminal inverted repeats.

Sequence analysis indicated that *B. orientalis* mt genome was arranged in a linear form. It containing three protein-coding genes, *cox1* (cytochrome *c* oxidase I), *cob* (cytochrome *b*), *cox3* (cytochrome *c* oxidase III) and six large subunit (LSU) rRNA gene fragments, but not any tRNA genes, which is consistent with those of other apicomplexan parasites studied to date (Figures 1 and 2) [11]. The mt genomes of apicomplexan parasites usually contain terminal inverted repeat (TIR) sequences with the size of around 440-450 bp [5]. However, in the mt genome of *B. orientalis*, 181 bp and 161 bp TIRs were identified from the beginning and the end, respectively (Figure 1). *cox1*, the first and fourth rRNA large subunit fragments (L1 and L4) are encoded by one strand of the mt genome, whereas *cox3*, *cob*, the second, the third, fifth and sixth fragments of large subunit rRNA genes (L2, L3, L5 and L6) are encoded by another strand (Figure 2a). The arrangement and predicted transcriptional direction of three protein-coding genes are the same as that of *B. bovis* (Figure 2b), *B. gibsoni* (not shown), and *Theileria parva* (Figure 2c), however, it greatly differed from that of *T. orientalis*, *T. equi* (not shown) and *P. falciparum* (Figure 2d) [5].

In this study, BLAST analysis of the entire mt genome sequence of *B. orientalis* to NCBI databases revealed that the mt genome sequence of *B. orientalis* was most similar to those of *B. bovis* (EU075182 and AB499088), with an identity of 87%. The other related species were *B. caballi*, *B. bigemina* and *B. gibsoni*, showing their mt genome sequence identities of 86%, 85% and 84% with that of *B. orientalis*, respectively. However, nucleotide identities were greatly different for the sequences of three protein-coding genes *cox1*, *cox3* and *cob*, when compared among different species. Pairwise comparison has been carried out among 13 different apicomplexan species including *B. orientialis*, another six *Babesia* species, five *Theileria* species and *P. falciparum* (see Table 3). The nucleotide sequence differences among 13 species ranged from 38.7% to 99.8% for *cox1* (Table 3a), from 28.8% to 92.5% for *cox3* (Table 3b) and 32.7% to 99.9% for *cob* (Table 3c), respectively. These results indicated that *cox1* and *cob* genes were more conserved than *cox3* gene. The different extent of conservation in the protein-coding genes suggested that mt genome sequences could be used as a gene marker for population evolutionary studies.

**Table 3:** **Nucleotide sequence identity (percentage) between *Babesiaorientalis *** **and 12 apicomplexan parasites**

**(a) Identity analyses based on the sequences of *cox*** **1**												
**Species**	**1**	**2**	**3**	**4**	**5**	**6**	**7**	**8**	**9**	**10**	**11**	**12**
1. *B. orientalis *(KF218819)												
2. *B. bovis *(EU075182)	80.6											
3. *B. bovis *(AB499088)	80.6	99.8										
4. *B. bigemina *(AB499085)	82.4	81.1	81.2									
5. *B. caballi *(AB499086)	82.2	80.9	81.1	85.0								
6. *B. gibsoni *(AB499087)	81.6	79.0	79.2	81.6	82.9							
7. *B. rodhaini *(AB624357)	68.4	67.0	67.0	66.3	67.5	69.5						
8. *T. parva *(Z23263)	73.9	71.2	71.1	72.7	72.8	74.3	66.5					
9. *T. parva *(AB499089)	74.8	72.1	72.0	73.6	73.7	75.2	67.4	98.6				
10. *T. equi *(AB499091)	38.9	39.3	39.3	38.9	39.0	41.0	38.7	39.1	39.3			
11. *T. orientalis *(AB499090)	74.0	72.1	72.1	73.0	73.5	74.6	66.9	78.1	79.1	38.0		
12. *T. annulata *(NT167255)	71.7	68.9	68.9	70.6	70.7	71.3	63.3	84.0	83.8	37.2	74.3	
13. *P. falciparum *(M76611)	61.1	60.9	60.9	60.2	60.2	62.1	61.5	59.6	60.1	36.0	60.0	58.0
**(b) Identity analyses based on the sequences of *cox3***												
1. *B. orientalis *(KF218819)												
2. *B. bovis *(EU075182)	80.9											
3. *B. bovis *(AB499088)	74.2	92.5										
4. *B. bigemina *(AB499085)	68.7	68.1	73.7									
5. *B. caballi *(AB499086)	65.6	67.2	72.4	77.6								
6. *B. gibsoni *(AB499087)	64.7	63.3	67.6	71.4	72.7							
7. *B. rodhaini *(AB624357)	34.4	34.6	34.2	33.0	32.2	32.2						
8. *T. parva *(Z23263)	53.1	52.3	47.5	47.5	49.3	49.4	28.3					
9. *T. parva *(AB499089)	54.1	53.7	56.8	56.8	59.0	59.1	31.6	83.5				
10. *T. equi *(AB499091)	29.6	29.8	29.7	28.8	29.3	31.8	32.8	29.5	33.3			
11. *T. orientalis *(AB499090)	33.0	34.3	34.4	35.8	32.8	34.3	51.6	28.4	31.8	33.0		
12. *T. annulata *(NT167255)	53.1	52.9	48.3	48.1	48.8	50.5	29.4	82.6	67.0	29.4	29.3	
13. *P. falciparum *(M76611)	45.8	44.4	42.3	43.5	43.2	46.6	31.7	40.6	43.2	32.5	32.5	41.3
**(c) Identity analyses based on the sequences of *cob***												
1. *B. orientalis *(KF218819)												
2. *B. bovis *(EU075182)	81.7											
3. *B. bovis *(AB499088)	81.7	99.9										
4. *B. bigemina *(AB499085)	81.0	80.3	80.4									
5. *B. caballi *(AB499086)	82.0	80.6	80.7	84.6								
6. *B. gibsoni *(AB499087)	78.1	77.1	77.0	78.2	78.5							
7. *B. rodhaini *(AB624357)	33.7	34.8	34.8	34.1	32.8	34.2						
8. *T. parva *(Z23263)	58.5	59.0	58.9	58.9	58.7	60.1	30.9					
9. *T. parva *(AB499089)	62.2	62.7	62.6	62.6	62.4	63.9	32.7	94.0				
10. *T. equi* (AB499091)	69.3	68.2	68.1	68.0	68.8	67.7	33.6	58.2	61.8			
11. *T. orientalis *(AB499090)	61.2	60.6	60.5	61.9	62.0	63.2	33.3	56.5	60.1	61.3		
12. *T. annulata *(NT167255)	62.7	62.2	62.1	62.6	62.5	64.1	32.9	68.8	73.1	61.7	60.6	
13. *P. falciparum *(M76611)	33.9	33.9	33.9	33.9	34.0	34.7	56.0	32.9	34.9	32.6	33.5	33.5

### Phylogenetic analysis

The majority of phylogenetic studies in the phylum apicomplexa have utilized 18S rRNA genes [27,42], which allow the analysis of the ancient relationships or strains and species differentiation by focusing on the highly conserved regions defining the critical secondary structure, or the more variable internal transcribed spacer regions, respectively. Because of different extent of conservation of three protein-coding genes and important function of mt genome, the extent of sequence difference might reflect the phylogenetic relationship across species of apicomplexan parasites. So the mitochondrion sequences may provide an alternative approach to conduct these studies [5,10,13].

**Figure 3:**
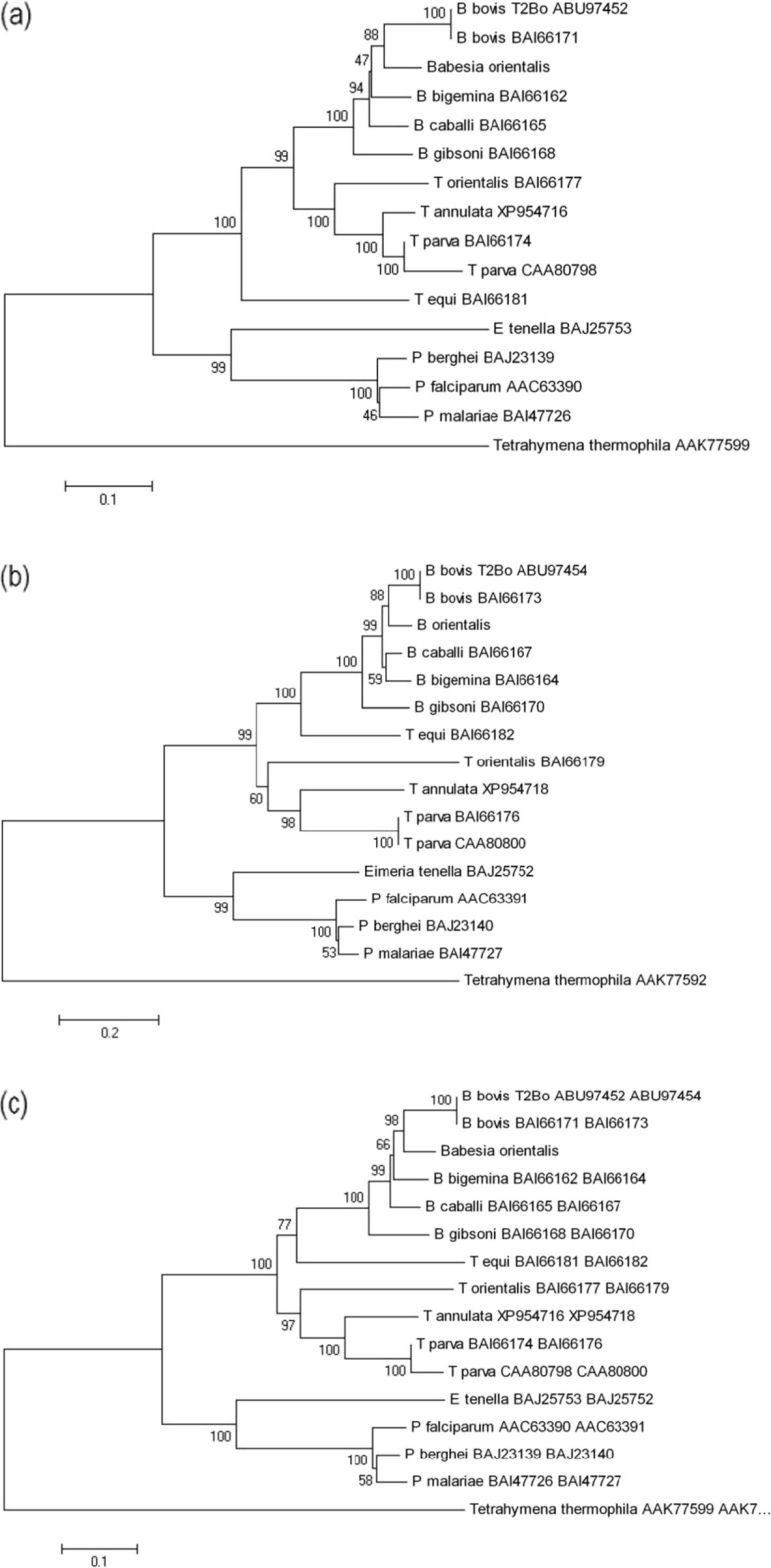
**Phylogenetic analyses of *cox*1 (a), *cob *(b) and *cox*1 +** ***cob *(c) amino acid sequences of *B. orientalis*.** Each neighbor-joining tree shows the phylogenetic relationship of protein coding genes of *Babesiaorientalis *mt genome determined in this study with other apicomplexan parasites. Bootstrap values at the nodes are indicating the degree of support for each cluster. Vertical lengths in each tree are not significant and are merely set for clarity. GenBank accession numbers are indicated on the right of each species name. *Tetrahymena thermophila* was used as an outgroup.

To analyze the phylogenetic relationship of *B. orientalis* with other apicomplexan parasites, phylogenetic trees were constructed with the amino acid sequences of *cox1*, *cob* and *cox1* + *cob* using neighbor-joining (NJ), maximum likelihood (ML), maximum parsimony (MP) and Bayesian phylogenetic methods. The trees obtained from three data sets by different methods were consistent with no significant changes in the topology or in the bootstrap values (Figures 3a,b, and c). The NJ trees constructed with *cox1*, *cob* and *cox1* + *cob* sequences have been presented as a representative (Figure 3). All three NJ trees displayed the same topology with high bootstrap values. *B. orientalis* appeared in the *Babesia* clade, and it’s most close to *B. bovis*. However, the bootstrap value was higher in the tree constructed by *cox1* + *cob* as compared to those of *cox1* and *cob*. These results suggested that the combined amino acid sequences of *cox1* and *cob* may be more reliable in studying evolutionary relationships than the sequences of single gene. The relationship of *B. orientalis* with other apicomplexan parasites revealed by all trees from mt genome sequences were consistent with that from the previous phylogenetic trees based on 18S rRNA and heat shock protein 70 (HSP70) gene [26,27]. These results demonstrated that the mt genome sequences are useful for the phylogenetic studies of apicomplexan parasites.

## Conclusion

In this study, we first reported the 5996 bp linear mitochondrion genome of *B. orientalis*. This mt genome contains 6 LSU rRNA gene fragments and three protein-coding genes, but no tRNA gene. Gene arrangement in the mt genome of *B. orientalis* is similar to those of *B. bovis*, *B. gibsoni*, and *T. parva*, but different from those of *T. orientalis*, *T. equi* and *P. falciparum*. Phylogenies based on the amino acid sequences of *cox1* or *cob* alone and *cox1* + *cob* combined all indicated that *B. orientalis* is closest to *B. bovis*, which is in agreement with the previous phylogenetic studies of *B. orientalis*. The availability of the entire mt genome sequences of *B. orientalis* provides valuable information for future phylogenetics, population genetics and molecular epidemiological studies of apicomplexan parasites.

## Competing interests

The authors declare that they have no competing interests.

## Authors’ contributions

All the authors contributed significantly to this study. LH and JLZ designed the experiments, carried out field and laboratory experiments, interpreted the results, and wrote the manuscript. YZ, QLZ, WJZ and HHF participated in cloning experiments and sequence analysis. MKK, MH and YQZ carried out editing and also helped in writing the manuscript. All the authors of the manuscript read and approved the final version of the manuscript.

## References

[B1] Frederick RL Shaw JM Moving mitochondria: establishing distribution of an essential organelle Traffic 2007 8 12 1668 1675 10.1111/j.1600-0854.2007.00644.x 17944806 PMC3739988

[B2] Taylor-Brown E Hurd H The first suicides: a legacy inherited by parasitic protozoans from prokaryote ancestors Parasit Vectors 2013 6 1 108 10.1186/1756-3305-6-108 23597031 PMC3640913

[B3] Kaczanowski S Sajid M Reece SE Evolution of apoptosis-like programmed cell death in unicellular protozoan parasites Parasit Vectors 2011 4 44 10.1186/1756-3305-4-44 21439063 PMC3077326

[B4] Gray G Maxwell D Villarimo A McIntosh L Mitochondria nuclear signaling of alternative oxidase gene expression occurs through distinct pathways involving organic acids and reactive oxygen species Plant Cell Rep 2004 23 7 497 503 10.1007/s00299-004-0848-1 15322810

[B5] Hikosaka K Watanabe Y-I Tsuji N Kita K Kishine H Arisue N Palacpac NMQ Kawazu S-i Sawai H Horii T Divergence of the mitochondrial genome structure in the apicomplexan parasites, *Babesia* and *Theileria* Mol Biol Evol 2010 27 5 1107 1116 10.1093/molbev/msp320 20034997

[B6] Feagin JE Mitochondrial genome diversity in parasites Int J Parasitol 2000 30 4 371 390 10.1016/S0020-7519(99)00190-3 10731561

[B7] Lung B Zemann A Madej MJ Schuelke M Techritz S Ruf S Bock R Hüttenhofer A Identification of small non-coding RNAs from mitochondria and chloroplasts Nucleic Acids Res 2006 34 14 3842 3852 10.1093/nar/gkl448 16899451 PMC1557801

[B8] Ward BL Anderson RS Bendich AJ The mitochondrial genome is large and variable in a family of plants (*Cucurbitaceae*) Cell 1981 25 3 793 803 10.1016/0092-8674(81)90187-2 6269758

[B9] Palmer DN Fearnley IM Walker JE Hall NA Lake BD Wolfe LS Haltia M Martinus RD Jolly RD Mitochondrial ATP synthase subunit c storage in the ceroid‒lipofuscinoses (Batten disease) American J Medical Genet 1992 42 4 561 567 10.1002/ajmg.1320420428 1535179

[B10] Hikosaka K Tsuji N Watanabe Y-I Kishine H Horii T Igarashi I Kita K Tanabe K Novel type of linear mitochondrial genomes with dual flip-flop inversion system in apicomplexan parasites, *Babesia microti* and *Babesia rodhaini* BMC Genomics 2012 13 1 1 9 10.1186/1471-2164-13-1 23151128 PMC3546061

[B11] Hikosaka K Watanabe Y-I Kobayashi F Waki S Kita K Tanabe K Highly conserved gene arrangement of the mitochondrial genomes of 23 *Plasmodium* species Parasitol Int 2011 60 2 175 180 10.1016/j.parint.2011.02.001 21329764

[B12] Boore JL Animal mitochondrial genomes Nucleic Acids Res 1999 27 8 1767 1780 10.1093/nar/27.8.1767 10101183 PMC148383

[B13] Lin R-Q Qiu L-L Liu G-H Wu X-Y Weng Y-B Xie W-Q Hou J Pan H Yuan Z-G Zou F-C Characterization of the complete mitochondrial genomes of five *Eimeria* species from domestic chickens Gene 2011 480 1 28 33 21402132 10.1016/j.gene.2011.03.004

[B14] Preiser P Wilson R Moore P McCready S Hajibagheri M Blight K Strath M Williamson D Recombination associated with replication of malarial mitochondrial DNA The EMBO J 1996 15 3 684 PMC449987 8599952

[B15] Lei R Shore GD Brenneman RA Engberg SE Sitzmann BD Bailey CA Kimmel LM Randriamampionona R Ranaivoarisoa JF Louis EE Jr Complete sequence and gene organization of the mitochondrial genome for Hubbard’s sportive lemur (*Lepilemur hubbardorum*) Gene 2010 464 1 44 49 20547216 10.1016/j.gene.2010.06.001

[B16] Hikosaka K Nakai Y Watanabe Y-I Tachibana S-I Arisue N Palacpac NMQ Toyama T Honma H Horii T Kita K Concatenated mitochondrial DNA of the coccidian parasite *Eimeria tenella* Mitochondrion 2011 11 2 273 278 10.1016/j.mito.2010.10.003 21047565

[B17] Hajibabaei M Singer GA Hebert PD Hickey DA DNA barcoding: how it complements taxonomy, molecular phylogenetics and population genetics Trends Genet 2007 23 4 167 172 10.1016/j.tig.2007.02.001 17316886

[B18] Fernando P Pfrender ME Encalada SE Lande R Mitochondrial DNA variation, phylogeography and population structure of the Asian elephant Heredity 2000 84 3 362 372 10.1046/j.1365-2540.2000.00674.x 10762406

[B19] Birungi J Munstermann LE Genetic structure of *Aedes albopictus* (Diptera: Culicidae) populations based on mitochondrial ND5 sequences: evidence for an independent invasion into Brazil and United States Ann Entomol Soc Am 2002 95 1 125 132 10.1603/0013-8746(2002)095[0125:GSOAAD]2.0.CO;2

[B20] Lewis MD Llewellyn MS Yeo M Acosta N Gaunt MW Miles MA Recent, independent and anthropogenic origins of *Trypanosoma cruzi* hybrids PLoS Neglect Trop Dis 2011 5 10 e1363 10.1371/journal.pntd.0001363 PMC3191134 22022633

[B21] Lee K-S Divis PC Zakaria SK Matusop A Julin RA Conway DJ Cox-Singh J Singh B *Plasmodium knowlesi*: reservoir hosts and tracking the emergence in humans and macaques PLoS Pathog 2011 7 4 e1002015 10.1371/journal.ppat.1002015 21490952 PMC3072369

[B22] Jongwutiwes S Putaporntip C Iwasaki T Ferreira MU Kanbara H Hughes AL Mitochondrial genome sequences support ancient population expansion in *Plasmodium vivax* Mol Biol Evol 2005 22 8 1733 1739 10.1093/molbev/msi168 15901839 PMC1224720

[B23] Liu ZL Ma LH Zhang GD Gao XS An investigation of babesiosis in buffaloes in Hubei province Acta Vet Zootechnica Sin 1986 17 1 49 54 (in chinese)

[B24] Liu ZL Ma LH Gao XS Cheng XJ Study on babesiosis of buffaloes in Hubei Province II: experimental infection demonstrated Rhipicephalus haemaphysaloides haemaphysaloides to be the vector of babesiosis in buffaloes Acta Vet Zootechnica Sin 1987 18 3 173 178 (in chinese)

[B25] Liu Z Zhao J Ma L Yao B *Babesia orientalis* sp. nov. parasitized in buffalo bubalus bubalis in China (Piroplasmida: Babesiidae) Acta Vet Zootechnica Sin 1997 28 1 84 89

[B26] He L Liu Q Quan M Zhou D-N Zhou Y-Q Zhao J-L Molecular cloning and phylogenetic analysis of *Babesia orientalis* heat shock protein 70 Vet Parasitol 2009 162 3 183 191 19375234 10.1016/j.vetpar.2009.03.039

[B27] Liu Q Zhao J Zhou Y Liu E Yao B Fu Y Study on some molecular characterization of *Babesia orientalis* Vet Parasitol 2005 130 3 191 198 15925722 10.1016/j.vetpar.2005.03.021

[B28] He L Zhou Y-Q Oosthuizen MC Zhao J-L Loop-mediated isothermal amplification (LAMP) detection of *Babesia orientalis* in water buffalo (*Bubalus babalis*, Linnaeus, 1758) in China Vet Parasitol 2009 165 1 36 40 19665847 10.1016/j.vetpar.2009.06.036

[B29] He L Feng H-H Zhang Q-L Zhang W-J Khan MK Hu M Zhou Y-Q Zhao J-L Development and evaluation of real-time PCR assay for the detection of *Babesia orientalis* in water buffalo (*Bubalus bubalis*, Linnaeus, 1758) J Parasitol 2011 97 6 1166 1169 10.1645/GE-2819.1 21711103

[B30] Katoh K Frith MC Adding unaligned sequences into an existing alignment using MAFFT and LAST Bioinformatics 2012 28 23 3144 3146 10.1093/bioinformatics/bts578 23023983 PMC3516148

[B31] Katoh K Standley DM MAFFT multiple sequence alignment software version 7: improvements in performance and usability Mol Biol Evol 2013 30 4 772 780 10.1093/molbev/mst010 23329690 PMC3603318

[B32] Carver T Harris SR Berriman M Parkhill J McQuillan JA Artemis: an integrated platform for visualization and analysis of high-throughput sequence-based experimental data Bioinformatics 2012 28 4 464 469 10.1093/bioinformatics/btr703 22199388 PMC3278759

[B33] Rutherford K Parkhill J Crook J Horsnell T Rice P Rajandream M-A Barrell B Artemis: sequence visualization and annotation Bioinformatics 2000 16 10 944 945 10.1093/bioinformatics/16.10.944 11120685

[B34] Katoh K Misawa K Kuma KI Miyata T MAFFT: a novel method for rapid multiple sequence alignment based on fast Fourier transform Nucleic Acids Res 2002 30 14 3059 3066 10.1093/nar/gkf436 12136088 PMC135756

[B35] Hall TA BioEdit: a user-friendly biological sequence alignment editor and analysis program for Windows 95/98/NT Oxford University Press 1999 41 95 98

[B36] Brunk CF Lee LC Tran AB Li J Complete sequence of the mitochondrial genome of *Tetrahymena thermophila* and comparative methods for identifying highly divergent genes Nucleic Acids Res 2003 31 6 1673 1682 10.1093/nar/gkg270 12626709 PMC152872

[B37] Posada D jModelTest: phylogenetic model averaging Mol Biol Evol 2008 25 7 1253 1256 10.1093/molbev/msn083 18397919

[B38] Swofford D PAUP 4.0 b10: phylogenetic analysis using parsimony 2002 Sunderland, MA, USA: Sinauer Associates

[B39] Huelsenbeck JP Ronquist F MRBAYES: Bayesian inference of phylogenetic trees Bioinformatics 2001 17 8 754 755 10.1093/bioinformatics/17.8.754 11524383

[B40] Ronquist F Huelsenbeck JP MrBayes 3: Bayesian phylogenetic inference under mixed models Bioinformatics 2003 19 12 1572 1574 10.1093/bioinformatics/btg180 12912839

[B41] Tamura K Dudley J Nei M Kumar S MEGA4: molecular evolutionary genetics analysis (MEGA) software version 4.0 Mol Biol Evol 2007 24 8 1596 1599 10.1093/molbev/msm092 17488738

[B42] Allsopp M Cavalier-Smith T DeWaal D Allsopp B Phylogeny and evolution of the piroplasms Parasitology 1994 108 147 147 10.1017/S0031182000068232 8159459

